# Atrial Fibrillation Ablation in Heart Failure and Preserved Ejection Fraction: An Observational Study of Risk Factors for Heart Failure Hospitalization

**DOI:** 10.2174/011573403X348844241129111639

**Published:** 2024-12-09

**Authors:** Rundi Qi, Hailei Liu, Yue Zhu, Nan Wu, Kexin Wang, Xiangwei Ding, Zhoushan Gu, Mingfang Li, Hongwu Chen, Weizhu Ju, Xin Li, Minglong Chen

**Affiliations:** 1 Department of Cardiology, The First Affiliated Hospital of Nanjing Medical University, Nanjing, China;; 2 Department of Cardiology, Taizhou School of Clinical Medicine, The Affiliated Taizhou People's Hospital of Nanjing Medical University, Nanjing Medical University, Taizhou, China;; 3 Department of Cardiology, Affiliated Hospital of Nantong University, Nantong, China;; 4 Department of Cardiology, Nanjing BenQ Medical Center, The Affiliated BenQ Hospital of Nanjing Medical University, Nanjing, China

**Keywords:** Atrial fibrillation, heart failure, radiofrequency catheter ablation, heart failure hospitalization, preserved ejection fraction, antiarrhythmic drugs, cardiovascular outcomes

## Abstract

**Introduction:**

Long-term heart failure hospitalization (HFH) after radiofrequency catheter ablation (RFCA) in atrial fibrillation (AF) patients with heart failure and preserved ejection fraction (HFpEF) and its risk factors remain to be investigated.

**Methods:**

AF patients with HFpEF who underwent RFCA from January, 2014 to December, 2018 from three centers were retrospectively included. Patients were assigned to the training and testing cohorts, respectively. In the training cohort, logistic regression analyses were performed to discriminate those with and without HFH. A scoring system was developed accordingly and validated.

**Results:**

A total of 417 AF patients with HFpEF receiving RFCA were enrolled. About 35 patients (8.4%) had HFH for 6 years. In the training cohort, the use of diuretics, atrial tachycardia (AT)/AF recurrence, prior HFH, and female sex were independent predictors of HFH in the multivariable analysis. A DAPF score (ranging from 0 to 9.0) was developed. The area under the receiver operating characteristic curve (AUC) of the DAPF score was 0.880 (95% CI, 0.830-0.929). A DAPF score ≥3.5 could predict HFH with a sensitivity of 81.8% and a specificity of 74.6%. The performance in the testing cohort remained robust (AUC, 0.858; 95% CI, 0.749-0.967).

**Conclusion:**

HFH in patients with AF and HFpEF after RFCA is not rare. The DAPF score could predict the risk of HFH in AF patients with HFpEF after RFCA and guide our treatment strategy.

## 1 INTRODUCTION

Atrial fibrillation (AF) and heart failure with preserved ejection fraction (HFpEF) are “vicious twins” with shared risk factors and clinical features, and they are inextricably linked to each other and contribute to increased morbidity, mortality, and adverse cardiovascular outcomes [1-3]. Previous studies reported that AF was present in 15-60% of HFpEF patients and was an independent predictor of death or heart failure hospitalization (HFH) [4-6]. In view of this, in addition to the treatment of HFpEF, AF control is essential for such a large population to interrupt this “vicious circle”. Currently, Radiofrequency Catheter Ablation (RFCA) is widely applied to treat symptomatic and drug-refractory AF patients [7-13]. In such a population with HFpEF, RFCA was also reported to be more effective in HFH reduction than antiarrhythmic drugs and even has the potential to reverse HFpEF [14-20]. Despite the above findings, HFH is still not rare even after RFCA [14, 15]. Thus, the prediction of HFH in AF patients with HFpEF after RFCA is essential for prognosis estimation and treatment strategy evaluation. However, limited data have been reported regarding predictors for HFH in AF patients with HFpEF post-RFCA [21-23]. Therefore, in this multicenter retrospective study, we aimed to study the long-term prognosis and develop a scoring system to predict HFH risk in such a population.

## 2 PATIENTS AND METHODS

### 2.1 Study Population

All AF patients with HFpEF aged 18-75 years old referred for RFCA between January 2014 and December 2018 were retrospectively enrolled from the First Affiliated Hospital of Nanjing Medical University, Affiliated Hospital of Nantong University, and Taizhou People’s Hospital. HFpEF was defined as follows: (1) NYHA class II-IV; (2) left ventricular ejection fraction (LVEF) ≥50%; (3) evidence of impaired diastolic function on echocardiography; and (4) N-terminal pro-brain natriuretic peptide (NT-proBNP) >125 (sinus rhythm) or >365 (AF) pg/ml [8, 24-26]. Patients without full medical records or with rheumatic heart disease, severe valvular heart disease (greater than mild stenosis, greater than moderate regurgitation), congenital heart disease, hypertrophic cardiomyopathy, dilated cardiomyopathy, severe renal dysfunction (an estimated glomerular filtration rate (GFR) of <30 ml per minute per 1.73 m^2^ of body-surface area or a serum creatinine level that was ≥2.5 mg per deciliter (221 μmol per liter)), and hyperthyroidism were excluded [27]. The study protocol adhered to the principles of the Declaration of Helsinki and was approved by the Institutional Review Board. All participants provided written informed consent.

### 2.2 Clinical Evaluation, Ablation, and Follow-up

The demographic data, medical histories, and medications of the patients were obtained from medical records systems. Before RFCA, blood samples were collected, and transthoracic echocardiography was performed. The modified Simpson method was used to measure LVEF. Anticoagulants were prescribed for at least 3 weeks, and antiarrhythmic drugs were discontinued for at least five half-lives before RFCA. Transesophageal echocardiography (TEE) was performed to exclude atrial thrombus. During the index procedure, a 3-dimensional left atrial geometry was constructed (Carto system or Ensite system), and circumferential pulmonary vein isolation (CPVI) was performed. Subsequent ablation beyond CPVI was performed at the discretion of the electrophysiologists [28-33]. Routine follow-ups were arranged at the 1^st^, 3^rd^, 6^th^, and 12^th^ months and then every year after RFCA. All patients received a clinical evaluation, electrocardiogram, transthoracic echocardiography, and 24-hour Holter monitoring during each follow-up period. In the case of any cardiovascular discomfort, a clinical evaluation was performed and recorded.

### 2.3 Event Definition and Evaluation

Hospitalization was defined as any in-hospital stay overnight. HFH was defined as any hospitalization for acute heart failure in which the patient needed extra diuretics or vasoactive drugs post RFCA, including intravenous medication for HF (diuretics, vasodilators, or inotropic agents), or who needed a substantial increase in oral diuretic therapy for HF (an increase of furosemide ≥40 mg or equivalent or the addition of a thiazide to a loop diuretic) [34-36]. Atrial tachycardia (AT)/AF recurrence was defined as any episode of atrial arrhythmia after a blanking period of 3 months post RFCA that lasted more than 30s [37-39]. All events were evaluated by an event committee consisting of three senior cardiologists independent of this study.

### 2.4 Statistical Analysis

Continuous variables were presented as the mean ± standard deviation (SD) if normally distributed and compared with Student’s t-test and as medians and interquartile range otherwise with the Mann‒Whitney U test. Categorical variables are described as counts and percentages and were compared with chi-square tests. Logistic regression was used to perform multivariate analysis. A two-sided *P* value of less than 0.05 was considered statistically significant. Analyses were performed with the SPSS statistical package, version 26 (IBM).

Patients from the First Affiliated Hospital of Nanjing Medical University and Taizhou People’s Hospital were assigned to the training cohort, and patients from another center were assigned to the testing cohort. In the training cohort, binary logistic regression was used to identify candidate variables that were significantly associated with HFH, and receiver operating characteristic (ROC) curves were analyzed to identify optimal cut points for discrimination, which were rounded to the nearest clinically significant integer when applicable. Thereafter, significant variables in the univariate analyses were entered into multivariable logistic regression models to determine a final model. Once the full multivariable model was created, stepwise backward elimination was performed with the least significant variable removed one at a time until all included model variables were statistically significant. The scoring system was developed according to the strength of association by β coefficients. The area under the curve (AUC) was used to analyze the diagnostic performance of the scoring system. Thereafter, the diagnostic performance of this scoring system was validated in the testing cohort.

## 3 RESULTS

### 3.1 Baseline Characteristics and Follow-up Results

**Fig. (1):**
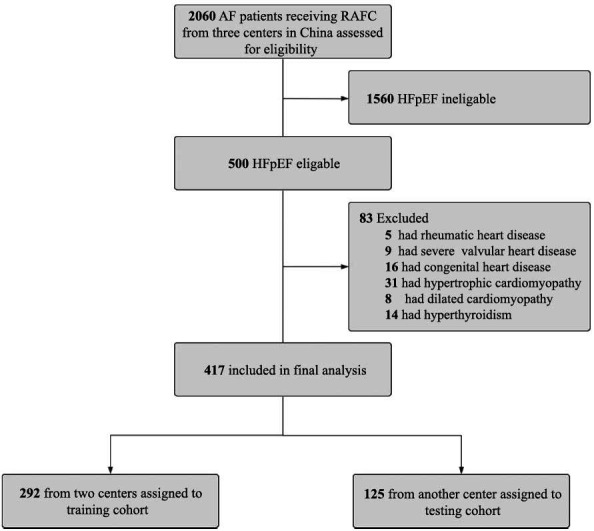
The algorithm of patient enrollment. **Abbreviations:** AF = atrial fibrillation; HFpEF = heart failure with preserved ejection fraction; RFCA = radiofrequency catheter ablation.

From January 2014 to December 2018, 2060 AF patients were referred for RFCA from three centers. Five hundred patients met the inclusion criteria, and 83 patients were excluded. Thus, 417 patients were included in the final analysis (Fig. **1**). During a median follow-up period of 6.0 ± 1.5 years, 35 patients (8.4%) had HFH. Patients from the First Affiliated Hospital of Nanjing Medical University and Taizhou People’s Hospital (n=292) were assigned to the training cohort, and patients from another center were assigned to the testing cohort (n=125). The baseline characteristics were balanced between the two cohorts (Table **1**).

### 3.2 Predictors of HFH

In the training cohort, 24 patients (8.2%) had HFH. The patients in the training cohort were divided into two groups according to whether they had HFH. The univariate analysis revealed that female sex, NYHA class, use of diuretics, prior HFH at baseline, and AT/AF recurrence were relevant to HFH (Table **2**).

After putting all these parameters into the multivariate analysis model, diuretic use (odds ratio=7.465; 95% CI: 1.694, 32.900, *P=*0.008), atrial tachycardia (AT)/AF recurrence (odds ratio=10.875; 95% CI: 3.566, 33.257; *P*<0.0001), prior HFH (odds ratio=5.460; 95% CI: 1.709, 17.441, *P=*0.004) and female sex (odds ratio=3.280; 95% CI: 1.127, 9.548, *P=* 0.029) were independently associated with HFH (Table **3**).

### 3.3 Derivation of the DAPF Score for HFH Prediction

**Fig. (2):**
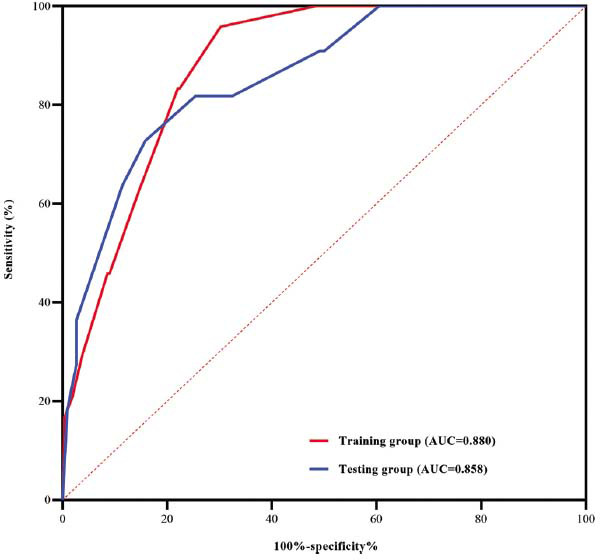
Receiver operating characteristics (ROC) curves of the D-AF score to predict HFH in the training and testing groups. The x-axis shows the false positive rate in the two groups for the D-AF score, and the y-axis shows the true positive rate in the two groups for the D-AF score. **Abbreviations:** AUC, area under the curve; HFH, heart failure hospitalization.

A DAPF score ranging from 0 to 9.0 points was derived from these 4 variables based on the strength of association with HFH in multivariate logistic regression (use of diuretics, 2.0 points; AT/AF recurrence, 3.0 points; female sex, 1.5 points; and prior HFH, 2.5 points). The DAPF score provided discrimination of HFH patients from controls with an AUC of 0.880 (Fig. **2**). A score of ≥3.5 points could predict HFH with a sensitivity of 81.8% and a specificity of 74.6% (*P*<0.001).

### 3.4 Validation of the DAPF Score in a Separated Testing Cohort

**Fig. (3):**
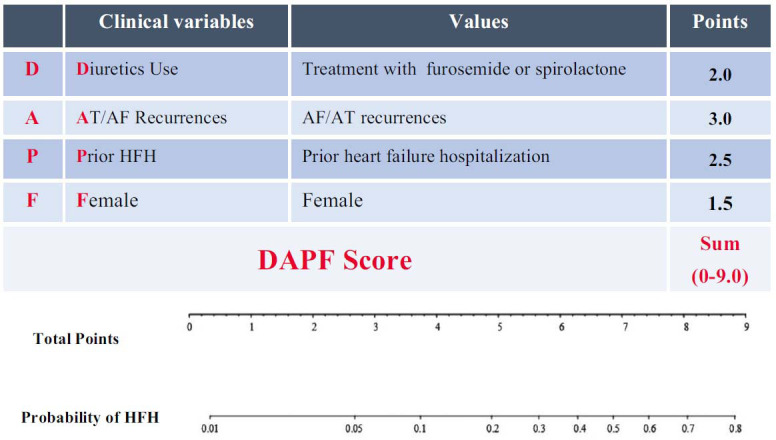
Description of the DAPF score. **Abbreviations:** AT = atrial tachycardia; AF = atrial fibrillation; HFH = heart failure hospitalization.

In the testing cohort, 11 patients (8.8%) had HFH. The baseline characteristics of the HF group and noHF group in the testing cohort are shown in Table **4**. The predictive value of the DAPF score was maintained with an AUC of 0.858 (95% CI, 0.749-0.967; Fig. **2**). A score of ≥3.5 points could predict HFH with a sensitivity of 83.3% and a specificity of 78.0% in the testing group (Fig. **3**).

## 4 DISCUSSION

The major findings of this study were as follows: (1) approximately 8.4% of AF patients with HFpEF suffered from HFH even after RFCA at a median follow-up of 6.0 years; (2) Diuretic use, atrial tachycardia (AT)/AF recurrence, prior HFH, and female sex were independent predictors for HFH in AF patients with HFpEF post-RFCA; and (3) the DAPF score could predict HFH risk in such a population. To the best of our knowledge, this is the first study to develop a scoring system to predict the long-term risk of HFH in AF patients with HFpEF after RFCA.

AF and HFpEF are two highly interacting diseases with shared pathophysiological backgrounds [40-43]. HFpEF is characterized by pathological increases in cardiac filling pressure at rest or under exertion. Left atrial dilation and electrical remodeling promote AF development [44-49]. Common risk factors and commodities, neurohumoral changes, systemic inflammation, left atrial remodeling, and electrophysiological alterations create a substrate for AF and HF to develop, either independently or concomitantly [46, 50]. RFCA was reported to be an effective way to interrupt this vicious cycle. In this study, we found that 8.4% of AF patients with HFpEF suffered from HFH even after RFCA, with a median follow-up of 6.0 years, and AT/AF recurrence was found in 22.5% of them. The results were similar to those of several other studies [22, 46, 51]. In view of this, although AF and HFpEF are vicious twins, their effects on patients could be fully or partially overlapped or even independent. Thus, even the same phenotype of AF with HFpEF might have different pathophysiological backgrounds and require different treatment strategies. Those phenotypes with an independent effect of AF and HFpEF on patients might benefit the least from RFCA alone than the other two types. Therefore, in the prediction of post-RFCA HFH in such a population, the models were significant in differentiating different types of AF with HFpEF in order to guide our clinical decision-making and prognosis evaluation.

The DAPF score system was derived from 4 variables that could be easily obtained from most patients, including diuretic use, AT/AF recurrence, prior HFH, and female sex. Among all these parameters, AT/AF recurrence had the strongest association with HFH, although some of the patients with HFH did not have AT/AF recurrence. A previous study indicated that restoration of sinus rhythm by ablation could reduce HFH in AF patients with HFpEF [52-54]. This finding is consistent with our results that AT/AF recurrence increased the HFH risk in AF and HFpEF patients. These results emphasize the crucial role of AF in HFpEF patients. As the major hallmark of HFpEF, AF could deteriorate diastolic dysfunction, which then aggravates symptoms and deteriorates the prognosis of HFpEF. Therefore, restoring sinus rhythm is crucial for these patients. However, it seemed that AT/AF was not accountable for all HFH in AF patients with HFpEF. In our study, we found some HFH patients without a recorded AT/AF recurrence and *vice versa*. AT/AF-contributed HFH could be resolved by successful ablation; however, residual risks remaining in HFpEF contributed to HFH, and HFH could still occur if HFpEF alone could lead to HF-related symptoms. Of note, other parameters, in addition to AT/AF recurrence in the DAPF score, were mostly representative of the severity of HFpEF, and this emphasizes the necessity of HFpEF control in such patients.

We found that female sex was associated with HFH in the univariate analysis. After the multivariable analysis, the female sex was still related to HFH. Although the mechanism of the association between female sex and HF remains unclear, the close relationship between left atrial function and hormone levels, as established in some studies, might serve as the major pathophysiological background [55-58].

Consistent with previous studies, prior HFH and diuretics therapy at baseline remained as robust risk factors for HFH in HFpEF with AF, even subsequent to ablation procedures [31, 59-62]. Notably, findings from the ENGAGE AF-TIMI 48 trial underscored the significant association between a history of HFH and the incidence of HF events, irrespective of HF phenotype. Additionally, a prior investigation highlighted congestion as the primary driver of HFH, necessitating escalated diuretic interventions [63-71]. Collectively, these factors intricately intertwine with the congestive state of patients, thus emerging as pivotal indicators for HFH occurrence.

## 5 LIMITATIONS

There are several limitations to this study. First, due to the retrospective observational nature, residual confounding, including the different operators, may potentially affect the results. However, the baseline characteristics were well balanced between the two cohorts, and the DAPF score derived from the training cohort is well tested in the independent testing cohort, rendering its generalizability. Second, the recurrence rate might be underestimated, as only regular 24-hour Holter monitoring was applied during follow-up. Third, the DAPF score was not prospectively verified. Although we tested in a cohort and performed external verification in another separate cohort, large prospective studies in diverse countries with a more heterogeneous population to assess the DAPF score system are warranted. In addition, the sample size was limited; multi-center and multi-area studies with more cases could further evaluate the efficacy of this score.

## CONCLUSION

HFH among patients with AF and HFpEF after RFCA is not rare. Diuretic use, atrial tachycardia (AT)/AF recurrence prior to HFH, and female sex were independent predictors of HFH. Furthermore, the DAPF score could predict the risk of HFH in AF patients with HFpEF after RFCA and guide our treatment strategy.

## Figures and Tables

**Table 1:** Baseline characteristics of AF patients with HFpEF after RFCA in the training and testing groups.

**Characteristics**	**Training Group (N=292)**	**Testing Group (N=125)**	***P* Value**
Age, year	60.4 ± 8.7	60.8 ± 8.4	0.705
Males	192 (65.8)	87 (69.8)	0.496
Body mass index, Kg/m^2^	25.1 ± 3.2	25.2 ± 3.4	0.781
Smoking	82 (28.1)	36 (28.8)	0.841
CHA2DS-VASc Score	1.7 ± 1.3	1.7 ± 1.3	0.985
HAS-BLED Score	1.0 ± 0.8	1.0 ± 0.9	0.980
PAF	120 (41.1)	44 (35.2)	0.275
AF symptom duration, years	5.1 ± 5.6	4.5 ± 4.2	0.066
AF recurrences	62 (21.2)	32 (25.6)	0.371
Heart rate, bpm	79.9 ± 15.0	79.6 ± 13.0	0.806
New York Heart Association Class			
II III IV	190 (65.1) 90 (30.8) 12 (4.1)	74 (59.2) 47 (37.6) 4 (3.2)	0.389
**Coexisting Medical Illnesses**			
Prior HFH	117 (40.1)	53 (42.4)	0.657
Hypertension	173 (59.2)	68 (54.4)	0.387
Coronary artery disease	45 (15.4)	18 (14.4)	0.801
Diabetes mellitus	38 (13)	12 (9.6)	0.411
Chronic obstructive pulmonary disease	1 (0.3)	1 (0.8)	0.510
Obstructive sleep apnea syndrome	6 (2.1)	3 (2.4)	0.824
Stroke/Transient ischemic attack	19 (6.5)	12 (9.6)	0.309
Bleeding	2 (0.7)	0 (0.0)	0.354
**Transthoracic Echocardiogram**			
Left ventricular ejection fraction, %	63.4 ± 3.3	63.3 ± 2.9	0.598
Left atrium diameter, mm	40.3 ± 4.7	40.5 ± 4.3	0.573
Left ventricular end diastolic dimension, mm	47.1 ± 3.9	47.3 ± 4.0	0.578
E/e'	9.0 ± 2.7	8.8 ± 2.2	0.919
**Blood Test**			
N-terminal pro-brain natriuretic peptide, pg/ml	786.3 ± 826.2	784.7 ± 847.1	0.986
Creatinine, umol/L	75.6 ± 17.5	74.0 ± 11.5	0.406
Hemoglobin, g/L	135.1 ± 16.6	136.5 ± 15.0	0.535
**Medications**			
Antiarrhythmic drugs	259(88.7)	113 (90.4)	0.731
Anticoagulants	279(95.5)	112 (97.6)	0.320
Antiplatelet	10(3.4)	1 (0.8)	0.185
ACEI/ARB	105 (36.0)	37 (29.6)	0.217
β block	119 (40.8)	45 (36.0)	0.383
Calcium channel blockers	47 (16.1)	19 (15.2)	0.884
Diuretic use	22 (7.5)	7 (5.6)	0.536
Digoxin	2 (0.7)	2 (1.6)	0.587
Glucose-lowering medication	38 (13)	12 (9.6)	0.411
Hydrochlorothiazide	6 (2.1)	5 (4.0)	0.317
Statin	58 (19.9)	23 (18.4)	0.788
Nitrates	7 (2.4)	6 (4.8)	0.222
Indapamide	0 (0.0)	1 (0.8)	0.300

**Note:** Values presented as mean±1 standard deviation for continuous variables and as n (%) for categorical variables. **Abbreviations:** ACEI/ARB = angiotensin-converting enzyme inhibitor/angiotensin receptor blocker; AF = atrial fibrillation; E/e’ = the peak velocities of the diastolic early (E) transmitral Doppler flow/the mitral annular velocities during early (e’)diastole; HFpEF = heart failure with preserved ejection fraction; PAF = paroxysmal atrial fibrillation; RFCA = radiofrequency catheter ablation.

**Table 2:** Baseline characteristics of patients with or without HFH in the training group.

**Characteristics**	**NoHFH Group (268)**	**HFH Group (24)**	***P* Value**
Age, year	60.29±8.758	61.71±8.024	0.446
Males	181 (67.5)	11 (45.8)	0.042*
Body Mass Index, Kg/m^2^	25.074±3.225	25.721±3.306	0.366
Smoking	75 (28.0)	7 (29.4)	0.808
CHA2DS-VASc Score	1.7±1.3	2.0±1.3	0.985
HAS-BLED Score	1.0±0.8	1.1±0.8	0.659
PAF	113 (42.2)	7 (29.2)	0.280
AF symptom duration, years	5.12±5.772	4.24±2.992	0.218
AF recurrences	49(18.3)	13(54.2)	0.0002*
Heart Rate, bpm	80.12±15.044	77.83±15.268	0.488
**New York Heart Association Class**			
II III IV	177 (66.0) 80 (29.9) 11 (4.1)	9 (37.5) 15 (62.5) 0 (0.0)	0.004*
**Coexisting Medical Illnesses**			
Prior HFH	98 (36.6)	19 (79.2)	0.000045*
Hypertension	158 (59)	15 (62.5)	0.830
Coronary artery disease	39 (14.6)	6 (25)	0.384
Diabetes mellitus	35 (13.1)	3 (12.5)	0.938
Chronic obstructive pulmonary disease	1 (0.4)	0 (0.0)	0.764
Obstructive sleep apnea syndrome	4 (1.5)	2 (8.3)	0.079
Stroke/Transient ischemic attack	19 (7.1)	0 (0.0)	0.382
Bleeding	2 (0.7)	0 (0.0)	0.671
**Transthoracic Echocardiogram**			
Left ventricular ejection fraction, %	63.51±3.342	62.58±3.035	0.189
Left atrium diameter, mm	40.14±4.684	41.63±4.261	0.135
Left ventricular end diastolic dimension, mm	47.04±3.925	47.21±3.451	0.840
E/e'	8.98±2.746	8.06±2.360	0.452
**Blood Test**			
N-terminal pro-brain natriuretic peptide, pg/ml	741.56±730.039	1258.37±1471.627	0.085
Creatinine, umol/L	75.207±16.483	79.336±26.135	0.571
Hemoglobin, g/L	135.46±15.749	131.6±23.667	0.390
**Medications**			
Antiarrhythmic drugs	237 (91.9)	22 (91.7)	0.827
Anticoagulants	258 (96.3)	23 (95.8)	0.617
Antiplatelet	9 (3.4)	1 (4.2)	0.582
ACEI/ARB	98 (36.6)	7 (29.2)	0.515
β Block	108 (40.3)	11 (45.8)	0.667
Calcium channel blockers	43 (16.0)	4 (16.7)	0.937
Diuretic use	14 (5.2)	8 (33.3)	0.0001
Digoxin	2 (0.7)	0 (0.0)	0.671
Glucose-lowering medication	35 (13.1)	3 (12.5)	0.938
Hydrochlorothiazide	5 (1.9)	1 (4.2)	0.405
Statin	54 (20.1)	4 (16.7)	0.796
Nitrates	7 (2.6)	0 (0.0)	0.423
Indapamide	0 (0.0)	0 (0.0)	-

**Note:** *P<0.05. Values presented as mean±1 standard deviation for continuous variables and as n (%) for categorical variables. Other abbreviations as in Table **1**.

**Table 3:** Univariate and multivariate analysis of parameters associated with HFH.

**Parameter**	**Univariate Analysis**	**Multivariable Logistic Regression**		
	***P* Value**	**β Estimate (Score)**	**OR (95% CI)**	***P* Value**
Diuretics use	0.0001	2.01 (2.0)	7.465 (1.694, 32.900)	0.008*
AT/AF Recurrence	0.0002	2.386 (2.5)	10.875 (3.566, 33.257)	0.000029*
Female Sex	0.042	1.188 (1.0)	3.280 (1.127, 9.548)	0.029*
NYHA class	0.004	1.013	2.753 (0.932, 8.126)	0.067
Prior HFH	<0.0001	1.697 (1.5)	5.460 (1.709,17.441)	0.004*

**Note:** *P<0.05. OR = odds ratio; CI = Confidence interval. Other abbreviations as in Table **1**.

**Table 4:** Baseline characteristics of patients with or without HFH in the testing group.

**Characteristics**	**NoHFH Group (114)**	**HFH Group (11)**	***P* Value**
Age, year	60.74±8.491	61.00±7.823	0.921
Males	83 (72.8)	4 (36.4)	0.033
Body mass index, Kg/m^2^	25.252±3.434	25.000±3.384	0.818
Smoking	34 (29.8)	1 (18.2)	0.621
CHA2DS-VASc Score	1.6±1.3	2.3±1.3	0.126
HAS-BLED Score	1.0±0.9	0.8±0.9	0.454
AF type (PAF n, %)	40 (35.1)	4 (36.4)	0.933
AF symptom duration, years	4.36±4.231	5.75±4.079	0.301
AF recurrences	24 (21.1)	8 (72.7)	0.001*
Heart Rate, bpm	79.78±13.357	77.18±9.39	0.530
**New York Heart Association Class**			
II III IV	74 (64.9) 37 (32.5) 3 (2.6)	4 (36.4) 5 (45.5) 2 (18.2)	0.019*
**Coexisting Medical Illnesses**			
Prior HFH	44 (38.6)	9 (81.8)	0.006*
Hypertension	63 (55.3)	5 (45.5)	0.546
Coronary artery disease	16 (14)	2 (18.2)	0.892
Diabetes mellitus	10 (8.8)	2 (18.2)	0.285
Chronic obstructive pulmonary disease	1 (0.9)	0 (0.0)	0.755
Obstructive sleep apnea syndrome	3 (2.6)	0 (0.0)	0.586
Stroke/Transient ischemic attack	11 (9.6)	1 (9.1)	0.952
Bleeding	0 (0.0)	0 (0.0)	-
**Transthoracic Echocardiogram**			
Left ventricular ejection fraction, %	63.25±3.045	63.27±1.679	0.984
Left atrium diameter, mm	40.46±4.366	41.27±4.221	0.558
Left ventricular end diastolic dimension, mm	47.38±4.056	46.36±3.557	0.426
E/e'	9.08±2.128	7.27±1.909	0.183
**Blood Test**			
N-terminal pro-brain natriuretic peptide, pg/ml	775.25±871.779	882.94±545.634	0.689
Creatinine, umol/L	73.987±11.293	73.843±13.914	0.975
Hemoglobin, g/L	137.32±14.897	125.8±36.7	0.156
**Medications**			
Antiarrhythmic drugs	103 (90.4)	9 (81.8)	0.376
Anticoagulants	109 (90.8)	11 (100)	0.626
Antiplatelet	1 (0.9)	0 (0.0)	0.755
ACEI/ARB	36 (31.6)	1 (9.1)	0.172
β block	40 (35.1)	5 (45.5)	0.523
Calcium channel blockers	18 (15.8)	1 (9.1)	0.555
Diuretic use	4 (3.5)	3 (27.3)	0.015
Digoxin	1 (0.9)	1 (9.1)	0.169
Glucose-lowering medication	10 (8.8)	2 (18.2)	0.285
Hydrochlorothiazide	5 (4.4)	0 (0.0)	0.478
Statin	19 (16.7)	436.4)	0.118
Nitrates	6 (5.3)	0 (0.0)	0.435
Indapamide	1 (0.9)	0 (0.0)	0.755

Note: *P<0.05. Values presented as mean±1 standard deviation for continuous variables and as n (%) for categorical variables. Other abbreviations are in Table **1**.

## Data Availability

The data supporting the finding of the study is available within the article.

## LIST OF ABBREVIATIONS

AF: Atrial Fibrillation
HFH: Heart Failure Hospitalization
HFpEF: Heart Failure and Preserved Ejection Fraction
NT-proBNP: N-terminal Pro-brain Natriuretic Peptide
RFCA: Radiofrequency Catheter Ablation
